# Do We Have Real Poverty in the United States of America?

**Published:** 2007-09-15

**Authors:** Paula Braveman

**Affiliations:** Center on Social Disparities in Health, University of California, San Francisco

Consider the images of starving children in Africa, Asia, or Latin America accompanying appeals for humanitarian aid. It is not difficult to understand why people deprived of the most basic material necessities for subsistence — adequate food, clean water, shelter from extreme heat or cold — would suffer high rates of preventable disease, disability, and premature death. Poverty in developing countries is often defined as living on less than $2.00 per person per day (1). By those terms, very few people in the United States would be poor. But poverty criteria for poor countries are not applicable in affluent countries with far higher living costs. The official U.S. poverty guideline in 2005 was an annual income of $19,350 for a family of four (2), which would represent wealth in many poor countries (3). Why, then, are *Preventing Chronic Disease* and other U.S. journals participating in this multi-journal issue, to be released October 22, 2007, on poverty and human development? Is it simply a magnanimous gesture to support fighting poverty and its adverse health consequences in poor countries, or is poverty an issue we must address at home?

In the days after Hurricane Katrina, televisions around the world revealed many desperate New Orleans residents, too poor to leave before the storm, who were without food, shelter, or clean water days later. The post-Katrina revelations were even more shocking because they exposed not only an inadequate disaster relief response, but also poverty and long-standing neglect of public services affecting poor African Americans that predated the hurricane. The depth of deprivation reached levels that many Americans had thought existed only in poor countries, not here. By official U.S. criteria, widely considered too low, more than one in eight individuals overall and one in five children younger than 5 years in the United States were poor in 2005 (2). We have the highest rates of poverty and child poverty among affluent nations (4). New Orleans' poverty was not an anomaly, but a reflection of widespread conditions in our country. Still, one might question: is U.S. poverty real, in the sense that it is associated with worse health? How could poverty affect one's health in a rich country?

In the now-famous Whitehall studies (5-7), British civil servants were categorized into several socioeconomic groups according to their occupational standing, ranging from unskilled manual workers at the bottom to the highest executives at the top. A stepwise gradient in morbidity and mortality rates over time was seen across the entire socioeconomic hierarchy, with health improving as position in the hierarchy rose. This result was surprising because none of the civil servants was poor in absolute terms, and all had free medical care. Even the professional/managerial group, just below the top executives, had worse health than the top group. The gradient persisted, although less markedly, after adjusting for smoking, diet, and exercise; thus, these behaviors could not entirely explain the socioeconomic gradient (5-7). What else could explain it?

The socioeconomic gradient in health has been observed across many different health outcomes, populations, and settings (5,8,9), including in the United States overall (10-13) and within different racial/ethnic groups (12). One explanation is that variations in health behaviors by income, education, and occupational standing (7,12) reflect differences in a range of socioeconomic resources in households and neighborhoods that can encourage and facilitate (or discourage and obstruct) healthier behaviors (14). For example, although racial discrimination can limit the benefits of higher income for some groups, higher income often permits one to live in an area that is safe and pleasant for exercising and is near markets selling healthy food. A higher-status job often means more control over one's work schedule and better transportation, permitting one to exercise, to shop for and cook healthy food rather than rely on fast food, and to find good childcare. Behaviors can explain some of the gradient, but they do not remove it (7,15). Access to and use of medical care also could contribute to the socioeconomic gradient in health (16,17).

Psychological factors also appear to be important to health outcomes (18). For example, higher income can mean less ongoing struggle to make ends meet and hence less ongoing stress (19). Chronic stress can lead to health damage through neuroendocrine, sympathetic nervous system, vascular, and immune pathways (20-22). Lack of control at work may be another piece of the puzzle explaining the socioeconomic gradient (23,24), along with psychological states associated with one's position in a social pecking order (25). Living in a highly unequal society may damage the health of everyone in it, not only the poor, at least in part through psychological phenomena (25). Poverty in childhood may be particularly harmful to health through both material and psychosocial pathways (26), with serious health consequences across the entire life course (27).

What are the implications for how we think about and address poverty in the United States? Poverty and "near poverty" (income up to twice the federal poverty guidelines) in the United States are prevalent and are associated with worse health outcomes among the population overall and among non-Hispanic whites and among blacks considered separately (12). These poor health outcomes may help explain the low ranking of the United States among affluent countries in life expectancy and infant mortality (4). But can anything be done in the United States? A full-time U.S. worker supporting a family of four on a minimum wage job is poor and will remain so even with proposed legislation on wages and taxes. We could directly reduce poverty by raising the minimum wage, the Earned Income Tax Credit, or both to levels that would lift working families out of poverty. However, not only do we have higher rates of poverty and child poverty and worse health indicators than other affluent nations but we also have weaker social safety nets. Social safety nets can reduce chronic stress among middle-class as well as low-income families even during good times by reducing worries about health insurance, childcare, educating one's children, and old-age pensions, and by limiting how far one can fall in hard times. We could strengthen social programs, such as childcare, education, and housing subsidies, along with community development efforts that reduce the impact of poverty. Social programs such as early childhood development interventions and good schools can indirectly but powerfully reduce poverty by resulting in higher educational attainment, which is linked to higher earnings, and education can help break the intergenerational transmission of poverty (28-30).

Real, health-damaging poverty affects a large proportion of the U.S. population and exacts an unacceptable toll in avoidable suffering, disability, and premature death. This toll also mean a less productive workforce and less economic growth. Current understanding of the damage caused by near poverty and absolute poverty underscores the urgency of addressing poverty at home as well as globally. Effective action will require addressing not only the obvious material and logistical hardships associated with low income but also the adverse psychological consequences of living and working conditions that create ubiquitous stress and disempowerment and weaken families and communities. Effectively addressing poverty can improve the health and well-being not only of the poor but also of the scores of millions of middle-class Americans who increasingly live in fear of slipping through the safety net (31).
